# Effect of Chronic Exercise Training on Blood Lactate Metabolism Among Patients With Type 2 Diabetes Mellitus: A Systematic Review and Meta-Analysis

**DOI:** 10.3389/fphys.2021.652023

**Published:** 2021-03-11

**Authors:** Tong Zhao, Shenglong Le, Nils Freitag, Moritz Schumann, Xiuqiang Wang, Sulin Cheng

**Affiliations:** ^1^Exercise, Health and Technology Centre, Department of Physical Education, Shanghai Jiao Tong University, Shanghai, China; ^2^Faculty of Sport and Health Science, University of Jyväskylä, Jyväskylä, Finland; ^3^Exercise Translational Medicine Centre, Shanghai Centre for Systems Biomedicine, Shanghai Jiao Tong University, Shanghai, China; ^4^School of Life Sciences and Biotechnology, Shanghai Jiao Tong University, Shanghai, China; ^5^Department of Molecular and Cellular Sport Medicine, Institute of Cardiovascular Research and Sport Medicine, German Sport University, Cologne, Germany; ^6^Olympic Training Center Berlin, Berlin, Germany

**Keywords:** sports medicine, hyperlactatemia, lactate threshold, lactic acid, physical training, T2DM

## Abstract

**Purpose:** To assess the effect of chronic exercise training on blood lactate metabolism at rest (i.e., basal lactate concentrations) and during exercise (i.e., blood lactate concentration at a fixed load, load at a fixed blood lactate concentration, and load at the individual blood lactate threshold) among patients with type 2 diabetes mellitus (T2DM).

**Methods:** PubMed (MedLine), Embase, Web of Science, and Scopus were searched. Randomized controlled trials, non-randomized controlled trials, and case-control studies using chronic exercise training (i.e., 4 weeks) and that assessed blood lactate concentrations at rest and during exercise in T2DM patients were included.

**Results:** Thirteen studies were eligible for the systematic review, while 12 studies with 312 participants were included into the meta-analysis. In the pre-to-post intervention meta-analysis, chronic exercise training had no significant effect on changes in basal blood lactate concentrations (standardized mean difference (SMD) = −0.20; 95% CI, −0.55 to 0.16; *p* = 0.28), and the results were similar when comparing the effect of intervention and control groups. Furthermore, blood lactate concentration at a fixed load significantly decreased (SMD = −0.73; 95% CI, −1.17 to −0.29; *p* = 0.001), while load at a fixed blood lactate concentration increased (SMD = 0.40; 95% CI, 0.07 to 0.72; *p* = 0.02) after chronic exercise training. No change was observed in load at the individual blood lactate threshold (SMD = 0.28; 95% CI, −0.14 to 0.71; *p* = 0.20).

**Conclusion:** Chronic exercise training does not statistically affect basal blood lactate concentrations; however, it may decrease the blood lactate concentrations during exercise, indicating improvements of physical performance capacity which is beneficial for T2DM patients' health in general. Why chronic exercise training did not affect basal blood lactate concentrations needs further investigation.

## Introduction

T2DM is a metabolic disease that is typically accompanied by changes in glucose metabolism (Gianatti et al., 2014). As an important substrate of glucose metabolism (Hui et al., 2017), lactate is a biomarker that may predict the incidence of diabetes independent of many other risk factors (Juraschek et al., 2013). Specifically, T2DM patients tend to exhibit higher basal blood lactate concentrations due to diminished oxidative capacity (Bruce et al., 2003; Crawford et al., 2010), reduced relevant monocarboxylate transporters (MCTs) (Juel et al., 2004), and diminished pyruvate dehydrogenase (PDH) (Avogaro et al., 1996). The basal lactate concentrations may be further augmented by metformin, which is considered the first-line drug for the treatment of T2DM (Adeva-Andany et al., 2014). Elevated basal blood lactate concentrations thereby may exaggerate insulin resistance (Choi et al., 2002; Wu et al., 2016) and affect glucose transport (Miller et al., 2002), all of which may deteriorate glucose control (Brinkmann and Brixius, 2015) and place an enormous burden on kidney function (Bellomo, 2002).

Chronic exercise training has been shown to improve oxidative capacity (Phielix et al., 2010; Zanuso et al., 2017), lactate transport (Opitz et al., 2014a,b; Opitz et al., 2015), and PDH activity (Nakai et al., 2002; Gudiksen et al., 2017) in T2DM patients, all of which may contribute to a normalized lactate metabolism. Previous studies have indicated that regular exercise leads to reduced blood lactate concentrations at fixed loads in T2DM patients (Dela et al., 1995; Eriksen et al., 2007; Mogensen et al., 2009; Scheede-Bergdahl et al., 2014; Brinkmann and Brixius, 2015), but it remains unclear whether this is true for basal blood lactate concentrations as well.

Interestingly, despite the immense clinical relevance of hyperlactatemia in T2DM patients, blood lactate concentrations are not commonly assessed in clinical practice. Moreover, to date, only one narrative review scientifically summarized the effects of chronic exercise training on blood lactate metabolism in T2DM patients and concluded that chronic exercise training is likely to help normalizing pathologic lactate metabolism (Brinkmann and Brixius, 2015). However, three gaps were identified in this work. First, no systematic literature search and pooled analysis were carried out. Second, by focusing on T2DM patients, no comparison to healthy controls was performed. Third, it was not distinguished between the various indices of blood lactate metabolism during exercises, such as blood lactate concentrations at a fixed load, load at a fixed blood lactate concentration, or load at the individual blood lactate threshold.

Therefore, with this systematic review and meta-analysis, we aimed to examine whether chronic exercise training can decrease basal blood lactate concentrations and blood lactate concentrations during exercise (by decreasing blood lactate concentration at a fixed load, increasing load at a fixed blood lactate concentration, or increasing load at the individual blood lactate threshold) in patients diagnosed with T2DM. We further aimed to explore possible differences regarding the effects of chronic exercise training on blood lactate metabolism between T2DM patients and healthy controls.

## Materials and Methods

This systematic review and meta-analysis was conducted in accordance with the preferred reporting items for systematic review and meta-analysis protocols (PRISMA-P) 2015 statement (Moher et al., 2015) and Cochrane Handbook (Green et al., 2008). The study protocol was registered in the international database of prospectively registered systematic reviews in health and social care (PROSPERO: CRD42020172616).

### Types of Outcome Measures

Two outcome measures were included in this systematic review. The primary outcome was the change in basal blood lactate concentrations, which was measured at a fasting state or before exercise training. As the secondary outcome, we assessed blood lactate concentrations during exercise, which included three variables: (1) blood lactate concentrations at a fixed load: blood lactate measured at a given fixed load (e.g., 30 Watts, 60 Watts) during an incremental exercise test; (2) load at a fixed blood lactate concentration: load corresponding to a fixed blood lactate concentration (e.g., 2, 4 mmol/L) obtained during an incremental exercise test, and (3) load at the individual blood lactate threshold: workload or oxygen uptake (VO_2_) corresponding to a rapid/distinct change in the inclination of the blood lactate curve (Faude et al., 2009).

Blood lactate concentrations were assessed from plasma or capillary finger blood samples, both of which have previously been shown to be highly correlated (*r* = 0.991) (Foxdal et al., 1990).

### Literature Search

The search strategy was structured according to the Peer Review of Electronic Search Strategies (PRESS) 2015 guidelines (McGowan et al., 2016). The electronic databases PubMed (MedLine), Embase, Web of Science, and Scopus were systematically searched for relevant studies until 19th January 2021. A combination of MeSH terms such as “humans and exercise with type 2 diabetes” were used with no restriction for the year of publication. The complete search strings are provided in Supplementary Table 1. Reference lists from previous relevant reviews and included studies were further examined as complementary sources.

### Eligibility Criteria and Study Selection

This systematic review included studies published in peer-reviewed scientific journals with English language. The detailed eligibility criteria followed the PICOS principle (i.e., population, intervention, comparison, outcome, and study design). Specifically, (1) The target population were patients diagnosed with T2DM. (2) The intervention consisted of chronic exercise training, defined as cumulative, structured and planned, repetitive bouts of physical activity lasting ≥4 weeks (Caspersen et al., 1985; Sandercock et al., 2005; Kenney et al., 2015). (3) Comparisons of baseline and post-intervention, exercise and non-exercise, and/or T2DM patients and healthy controls were conducted. (4) The studies were required to report basal blood lactate concentrations and/or blood lactate concentrations during exercise (i.e., blood lactate concentrations at a fixed load, load at a fixed blood lactate concentration, or load at the individual blood lactate threshold). (5) Only original longitudinal intervention studies (i.e., randomized controlled trials (RCTs), non-randomized controlled trials (NRCTs), and case-control study) were included, while cross-sectional designs or cohort studies were excluded.

**Figure 1:**
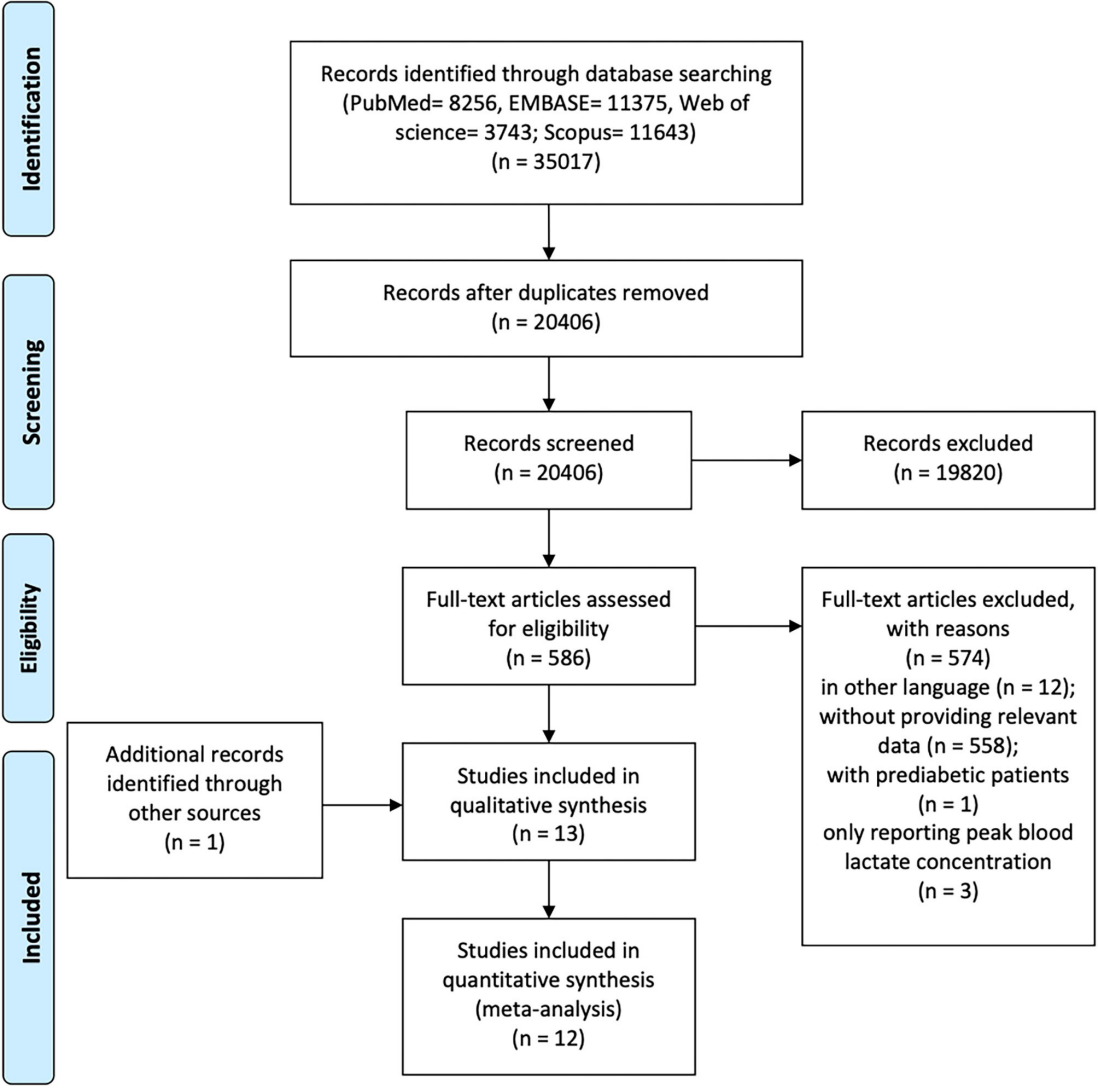
PRISMA flowchart of the systematic review process. PRISMA, Preferred Reporting Items for Systematic Reviews and Meta-Analysis.

Initially, the search records were imported to Endnote X9 software (Endnote, Clarivate Analytics, Stanford, Connecticut, USA) and duplicates were removed. Next, the titles and abstracts of identified articles were checked for relevance separately by 2 reviewers (T.Z. and S.L.). Subsequently, the reviewers independently reviewed the full texts of potentially eligible articles. Any disagreements were discussed with a third reviewer (S.C.) until a consensus was achieved. Figure 1 presents the flowchart of the study selection process.

### Data Extraction

The authors, publication year, study design, outcome variables, the testing protocol for outcomes, participant characteristics, and exercise protocol were extracted and summarized in Table 1. Where possible, the medication status of participants was extracted and summarized in Supplementary Table 2.

**Table 1:** Overview of included studies.

**No**.	**References**	**Study design**	**Study quality**	**Outcomes**	**Testing protocol for blood lactate concentration during exercise**	**Participants describe (*N*): %male, age, BMI, HbA1c**	**Exercise protocol**
1	Schneider et al. (1987)	Case-control	Fair	Basal blood lactate concentrations (following an overnight fast and 72 h after the last exercise bout)		**T2DM (20):** 100, 51, 78.2^‡^, 12.2 **Healthy control (11):** 100, 46, 81.6^‡^, 7.7	6 weeks of supervised aerobic training; 3 times per week for 30 min; intensity was at 50–70% VO_2_max^§^
2	Dela et al. (1995)	Case-control	Fair	Blood lactate concentrations at a fixed load (60 Watts)	One-legged graded bicycle test (cycle ergometer; until exhaustion)	**T2DM (7):** 100, 58, 29.1, 7.6 **Healthy control (8):** 100, 59, 25.7	10 weeks of home-based one-legged ergometer bicycling; 6 times per week for 30 min; intensity was at 70% VO_2_max
3	Holton et al. (2003)	Case-control	Fair	Load at the individual blood lactate threshold	Continuous graded test (cycle ergometer; starting at a cadence of 50 rpm at resistance of 0 or 20 Watts (for male or female, respectively) and increasing 20 Watts per 3 min until subjects' volitional fatigue)	**T2DM (9):** 77.8, 55, 35.0, 7.1 **Healthy control (10):** 70.0, 57, 32.3, 5.7	10 weeks of supervised moderate aerobic training (cycling, walking, and other forms); 3 times per week for 20–45 min; intensity was at 50–65% HR reserve
4	Juel et al. (2004)	Case-control	Fair	Basal blood lactate concentrations (following an overnight fast and 24 h after the last exercise bout)		**T2DM (10):** 100, 62, 28.3, 7.4 **Healthy control (7):** 100, 61, 24.5, 6.0	6 weeks of supervised strength training on single leg; 3 times per week for no more 30 min; 3–4 sets of 8–12 repetitions utilizing 50–100 RM from week 1 to 6
5	Baum et al. (2007)	RCT	Fair	Load at a fixed blood lactate concentration (4 mmol/L)	Incremental test (cycle ergometer; increasing every 3 min for 25 Watts until blood lactate concentration exceeded 4 mmol/L)	**Strength (13):** 60, 63, 86.5^‡^, 6.8	12 weeks of strength training on main muscles of the upper and lower body, 3 times per week for about 45 min; 1–3 sets with 12 repetitions at 70–80% of 1 RM
						**Vibration (14):** 60, 62, 83.3^‡^, 7.3	12 weeks of vibration exercise on a horizontal swinging platform with an amplitude of 2 mm; 3 times per week for about 20 min; vibration frequency was set at 30–35 Hz
6	Eriksen et al. (2007)	RCT	Fair	Blood lactate concentration at a fixed load (90 Watts)^†^	Submaximal exercise test (cycle ergometer; 8 min duration with incrementally increasing workloads)	Group 1*30 (9): 100, 59, 30, 7.5	4–5 weeks of home-based single long-duration exercise (a moderate to high intensity on an ergometer bike); 6 times per week for 30 min; intensity was at 60–65% VO_2_max
						Group 3*10 (9): 100, 60, 35, 7.6	4–5 weeks of home-based multiple short-duration exercise (a moderate to high intensity on an ergometer bike); 6 times per week for 10 min*3; intensity was at 60–65% VO_2_max
7	Michishita et al. (2008)	Case-control	Fair	Load at the individual blood lactate threshold	Submaximal exercise test (cycle ergometer; increasing every 4 min depending on their daily activity levels until blood lactate concentration exceeded 4 mmol/L)	**T2DM (10):** 30, 59, 30.2, 6.8 **Healthy control (10):** 20, 51, 29.7, 5.2	12 weeks of low intensity exercise therapy; 30–60 min per day; 1–6 times per week; intensity was at lactate threshold
8	Mogensen et al. (2009)	Case-control	Good	Load at a fixed blood lactate concentration (4 mmol/L)Blood lactate concentrations at fixed loads (30, 60, 90, 120, 150 Watts)^†^	Submaximal exercise test (cycle ergometer; after 4 min warm-up, beginning at 30 Watts, increasing by 30 Watts every 4 min until respiratory exchange rate value reached 1.0)	**T2DM (12):** 100, 53, 33.5, 7.3 **Healthy control (11):** 100, 56, 33.2, 5.3	10 weeks of combined interval training and continuous training; 2 times per week was under supervised and 2–3 times was at home; 20–30 min per session; intensity was at 50–70%VO_2max_
9	Opitz et al. (2014a)	NRCT	Fair	Load at fixed blood lactate concentrations (2, 4 mmol/L)	Endurance test (cycle ergometer; starting at 25 Watts, increasing 25 Watts every 2 min until exhaustion)	**Exercise (8):** 100, 56, 31.7, 6.7 **Non-exercise (15):** 100, 58, 32.4, 6.6	12 weeks of supervised endurance training on endurance; 3 times per week for 25–50 min; intensity was at 65–75% VO_2peak_
10	Scheede-Bergdahl et al. (2014)	Case-control	Poor	Basal blood lactate concentrations (before exercise test)Blood lactate concentrations at fixed loads (40, 60, 80 Watts)	Maximal exercise test (rowing ergometer; specific protocol was not shown)	**T2DM (12):** 100, 60, 29.1, 7.7 **Healthy control (9):** 100, 52, 31.5, 5.4	8 weeks of homebased aerobic training on a rowing ergometer; 3–4 times per week for 30 min; intensity was at 65–70% VO_2peak_
11	Opitz et al. (2015)	NRCT	Good	Basal blood lactate concentrations (before exercise test)Load at fixed blood lactate concentrations (2, 4 mmol/L)	Endurance test (cycle ergometer; starting at 25 Watts, increasing 25 Watts every 2 min until exhaustion)	**Exercise (10):** 100, 61, 30.6, 6.8 **Non-exercise (9):** 100, 54, 31.7, 6.9	12 weeks of supervised endurance training on ergometer; 3 times per week for 25–50 min; intensity was at 65–75% VO_2peak_
12	Støa et al. (2017)	NRCT	Good	Load at the individual blood lactate threshold	Incremental test (treadmill; an incline of 3%; consisting of 3 or 4 submaximal workloads each lasting 5 min; starting at 60% VO_2max_, speed was increased per 5 min until the subjects elicited an intensity above lactate threshold defined as the warm up [La^−^]_b_ value +2.3 mmol/L)	**MIT (19):** 100, 59, 31.1, 6.8	12 weeks of supervised moderate-intensity training (walking); 3 times per week for 60 min; intensity was at 73%HR_peak_
						**HAIT (19):** 100, 69, 32.0, 7.8	12 weeks of supervised high-intensity aerobic interval training (walking or running uphill); 3 times per week; 4*4 min at was 85–95% HR_peak_ and 36 min at 70% HR_peak_.
13	de Sousa et al. (2019)	RCT	Fair	Basal blood lactate concentrations (following an overnight fast)		**Exercise (19):** 52.6, 61, 33.0, 7.3 **Non-exercise (22):** 45.4, 61, 32.7, 7.3	12 weeks of supervised soccer training; 3 times per week for about 40 min; intensity at 50–100% HR_max_^§^.

*BMI, body mass index; HAIT, high-intensity aerobic interval training; HbA1c, hemoglobin A1c; HR, heart rate; MIT, moderate-intensity training; NRCT, non-randomized controlled trial; RCT, randomized controlled trial; RPE, rated perceived exertion; RM, repetition maximum; VO_2_, oxygen consumption*.

† *result excluded from qualitative synthesis due to the lack of original data*.

‡ *body weight instead of BMI, since the BMI was not given*.

§ *combined with dietary intervention*.

Outcome variables at baseline and post-intervention were extracted as mean and standard deviation (SD) from all intervention and control groups, as suggested by the Cochrane Collaboration Handbook (Green et al., 2008). Established methods (Green et al., 2008; Wan et al., 2014) were used if the mean or SD was not reported in the original article, otherwise, the study authors were contacted directly for the original data. For two studies that reported both plasma and erythrocyte lactate concentrations (Opitz et al., 2014a, 2015), only the plasma lactate values were extracted. One study (Støa et al., 2017) that measured blood lactate thresholds with two methods [i.e., velocity and percentage of maximal oxygen uptake (%VO_2max_)], both of values were extracted, while %VO_2max_ as a more commonly used method was analyzed for main outcomes.

### Quality Assessment and Risk of Bias

Two reviewers (T.Z. and S.L.) independently assessed the quality of included studies using Downs and Black checklist (Downs and Black, 1998). The checklist is customized to assess the methodological quality both of randomized and non-randomized studies of health care interventions. Downs and Black checklist is a 27-item scale with 5 subscales (i.e., reporting, external validity, bias, confounding, and power), and the sum score is 31 points. In line with another published systematic review (Korakakis et al., 2018), the last item (Q27, ranged from 0 to 5 points) was modified as “0 or 1 point” - “*whether the study performed a power calculation (Yes, 1 point), or missed a power calculation (No, 0 point)*.” Therefore, the revised sum score of the checklist is 28 points. The study quality was then classified as excellent (26–28), good (20–25), fair (15–19), and poor (≤14) (Korakakis et al., 2018). Any disagreements were discussed with a third reviewer (S.C).

### Statistical Analyses

All statistical analyses were performed using R (RStudio V3.6.1, Boston, Massachusetts, USA), with the *meta* package. standardized mean differences (SMD) with 95% confidence intervals (95% CI) were calculated as effect sizes (Martinez-Alonso et al., 2017). Hedges' *g* was used to correct the potential bias of SMD, due to the small sample sizes of included studies (Littell et al., 2008). A random effects model was used since it calculates the effect size based on inverse variance and between-study variance (Littell et al., 2008). The effect size was categorized as small (0.2), medium (0.5), and large (0.8) based on the absolute value of SMD (Littell et al., 2008). The heterogeneity was tested with *Q*-statistic and assessed with the *I*^2^ index and *p*-value. The *I*^2^ index of 25, 50, and 75% indicated low, medium, and high levels of heterogeneity, respectively (Green et al., 2008).

The effects of chronic exercise training were analyzed in three ways. (1) Within-group analysis was performed in all exercising T2DM groups by estimating the changes from baseline to post-intervention. (2) Between-group analysis was performed to compare the difference in relative changes between exercise and non-exercise groups in controlled experiments. (3) For case-control studies, differences in relative changes between T2DM groups and healthy control groups were calculated. Additionally, for “within-group analysis,” sensitivity analysis was conducted to test the robustness of results by removing studies which combined exercise with dietary interventions (Green et al., 2008). To detect whether methodological differences in measurements of basal blood lactate concentration (i.e., at fasting state or before exercise) affect the results, subgroup-analysis was performed. Additionally, to examine the bias of measurement methods for load at the individual blood lactate threshold, a sensitivity analysis was performed by using the velocity instead of %VO_2max_ at the blood lactate threshold. Meta-regression, and tests for funnel plots asymmetry were not performed due to a limited number of included studies (i.e., <10 for each variable) (Green et al., 2008).

Two reviewers (T.Z. and S.L) independently assessed the certainty of evidence for main outcomes using the Grading of Recommendations Assessment, Development, and Evaluation (GRADE) system (Guyatt et al., 2011). This system categorizes the quality of the total body of evidence in four level (i.e., “High,” “Moderate,” “Low,” and “Very Low”). First, RCTs start as high-quality evidence and observational studies as low-quality evidence. Second, several factors were evaluated. For example, five factors (i.e., risk of bias, imprecision, inconsistency, indirectness, publication bias) might lead to downgrade the level of evidence, and four factors (i.e., large magnitude of effect, dose response, and confounders likely) might lead to upgrade the level. In particular, the overall risk of bias (i.e., risk of bias for an outcome across studies) was evaluated according the Cochrane Handbook and the established standard for levels of evidence (Green et al., 2008; Oosterhoff et al., 2019).

## Results

The flowchart of study selection is presented in Figure 1. A total of 35,017 articles were initially identified. After screening, 13 studies were included in the qualitative synthesis and 12 studies in the meta-analysis. One study (Eriksen et al., 2007) was excluded from the meta-analysis due to insufficient reporting (e.g., only with log-transformed data). The GRADE evidence profiles for main outcomes were showed in Supplementary Table 3.

### Description of Included Studies

Study characteristics are summarized in Table 1. The 13 included studies were published from 1987 to 2019, including three RCTs (Baum et al., 2007; Eriksen et al., 2007; de Sousa et al., 2019), three NRCTs (Opitz et al., 2014a, 2015; Støa et al., 2017), and seven case-control studies (Schneider et al., 1987; Dela et al., 1995; Holton et al., 2003; Juel et al., 2004; Michishita et al., 2008; Mogensen et al., 2009; Scheede-Bergdahl et al., 2014). Three studies (Mogensen et al., 2009; Opitz et al., 2015; Støa et al., 2017) were assessed as “good” quality, nine studies (Schneider et al., 1987; Dela et al., 1995; Holton et al., 2003; Juel et al., 2004; Baum et al., 2007; Eriksen et al., 2007; Michishita et al., 2008; Opitz et al., 2014a; de Sousa et al., 2019) were of “fair” quality, and one study (Scheede-Bergdahl et al., 2014) was rated with “poor” quality. The detailed quality assessment of studies can be found in Supplementary Table 4. Among the included studies, five studies (Schneider et al., 1987; Juel et al., 2004; Scheede-Bergdahl et al., 2014; Opitz et al., 2015; de Sousa et al., 2019) assessed basal blood lactate concentrations, four studies (Schneider et al., 1987; Holton et al., 2003; Eriksen et al., 2007; de Sousa et al., 2019) assessed blood lactate concentrations at a fixed load, four studies (Baum et al., 2007; Mogensen et al., 2009; Opitz et al., 2014a, 2015) assessed load at a fixed blood lactate concentration, and three studies (Holton et al., 2003; Michishita et al., 2008; Støa et al., 2017) assessed the load at the individual blood lactate threshold (see Table 1).

A total of 312 participants were included. Among them, 200 participants were in exercising T2DM groups, 46 participants were in non-exercise T2DM groups, and 66 participants were healthy controls. Exercise protocols included continuous aerobic training (Schneider et al., 1987; Dela et al., 1995; Holton et al., 2003; Eriksen et al., 2007; Michishita et al., 2008; Opitz et al., 2014a, 2015; Scheede-Bergdahl et al., 2014; Støa et al., 2017), interval aerobic training (Støa et al., 2017), strength training (Juel et al., 2004; Baum et al., 2007), combined interval and continuous training (Mogensen et al., 2009), multiple short-duration training (i.e., three 10 min sessions per day) (Eriksen et al., 2007), vibration training (Baum et al., 2007), and soccer training (de Sousa et al., 2019). The exercise duration ranged from 4 weeks to 12 weeks and exercise frequency ranged from 1 to 6 times per week (see Table 1).

### Effects of Chronic Exercise Training on Basal Blood Lactate Concentration

**Figure 2:**
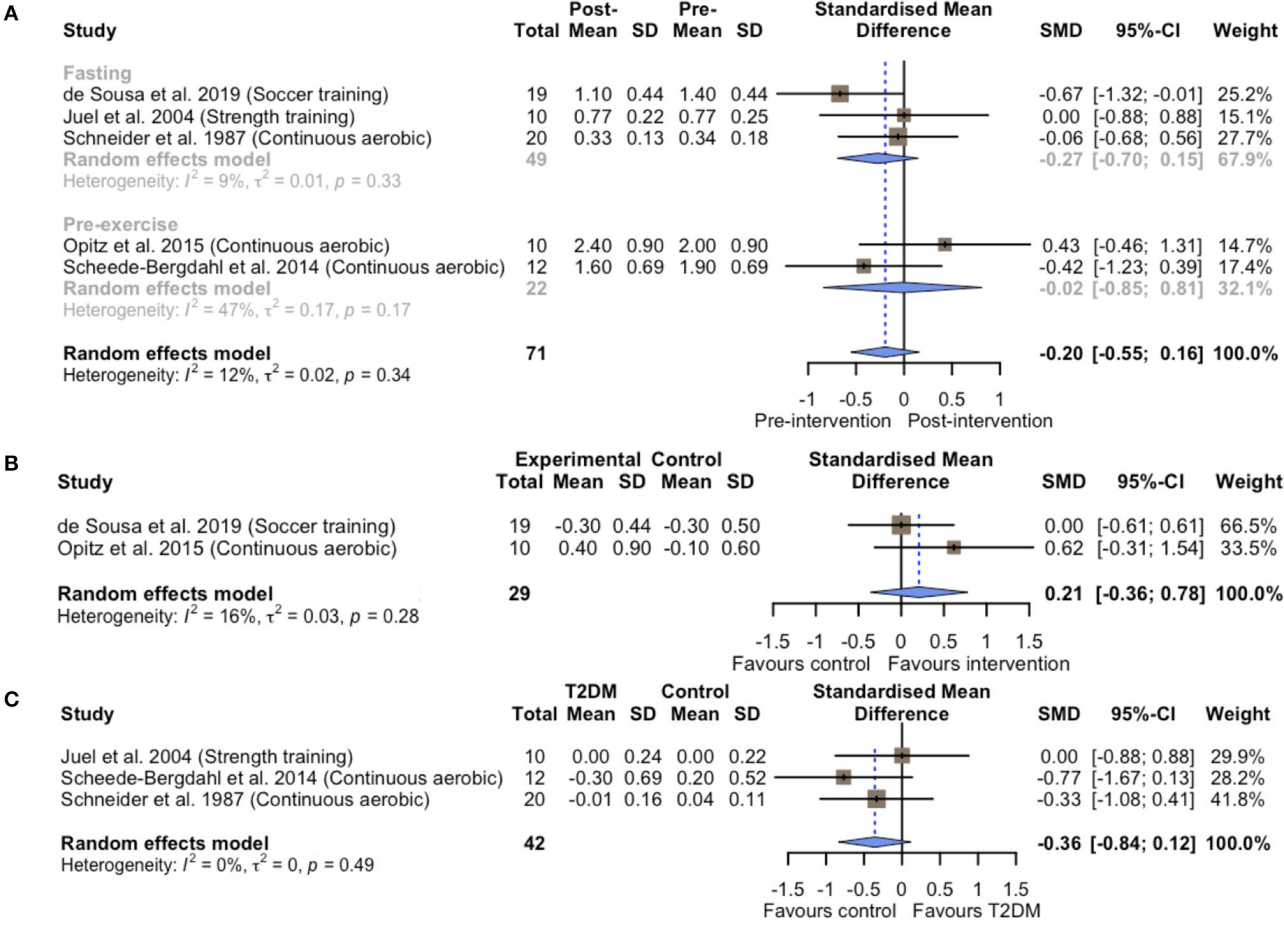
Forest plots of the effect of chronic exercise training on basal blood lactate concentrations **(A)** within-group analysis; **(B)** between-group analysis; **(C)** difference in relative change between T2DM patients and healthy controls.

Within-group comparisons consisted of five exercise groups including 71 T2DM patients. No changes were observed in basal blood lactate concentrations following different types of exercise training (SMD = −0.20; 95% CI, −0.55 to 0.16; *p* = 0.28; *I*^2^ = 12%; Figure 2A). A subsequent sensitivity analysis excluding two studies that used combined exercise and dietary interventions (Schneider et al., 1987; de Sousa et al., 2019) resulted in similar non-significance (SMD = −0.02; 95% CI, −0.52 to 0.47; *p* = 0.92; *I*^2^ = 0%; Supplementary Figure 1). Similarly, the between-group analysis on basal blood lactate concentrations for two controlled experiments (Opitz et al., 2015; de Sousa et al., 2019) showed no statistical differences between the exercise and non-exercise groups after the intervention (SMD = 0.21; 95% CI, −0.36 to 0.78; *p* = 0.48; *I*^2^ = 16%; Figure 2B). The results of subgroup-analysis showed that chronic exercise had non-significant effects on both the different measures of basal lactate concentrations in a fasting state (SMD = −0.27; 95% CI, −0.70 to 0.15; *p* = 0.20; *I*^2^ = 9.4%) or before exercise (SMD = −0.02; 95% CI, −0.85 to 0.81; *p* = 0.97; *I*^2^ = 47.3%).

To confirm whether these non-significant results observed above were due to T2DM initially exhibiting higher basal blood lactate levels than the healthy controls, we further compared the effects of chronic exercise training on basal blood lactate concentrations between T2DM and healthy control groups in three case-control studies (Schneider et al., 1987; Juel et al., 2004; Scheede-Bergdahl et al., 2014). Indeed, T2DM patients appeared to exhibit higher basal blood lactate concentrations at baseline compared to the healthy controls (SMD = 0.72; 95% CI, 0.18 to 1.26; *p* = 0.01; *I*^2^ = 16%; Supplementary Figure 2). However, the blood lactate concentrations were maintained both in T2DM patients and healthy controls (SMD = −0.36; 95% CI, −0.84 to 0.12; *p* = 0.14; *I*^2^ = 0%; Figure 2C).

### Effects of Chronic Exercise Training on Blood Lactate Metabolism During Exercise

**Figure 3:**
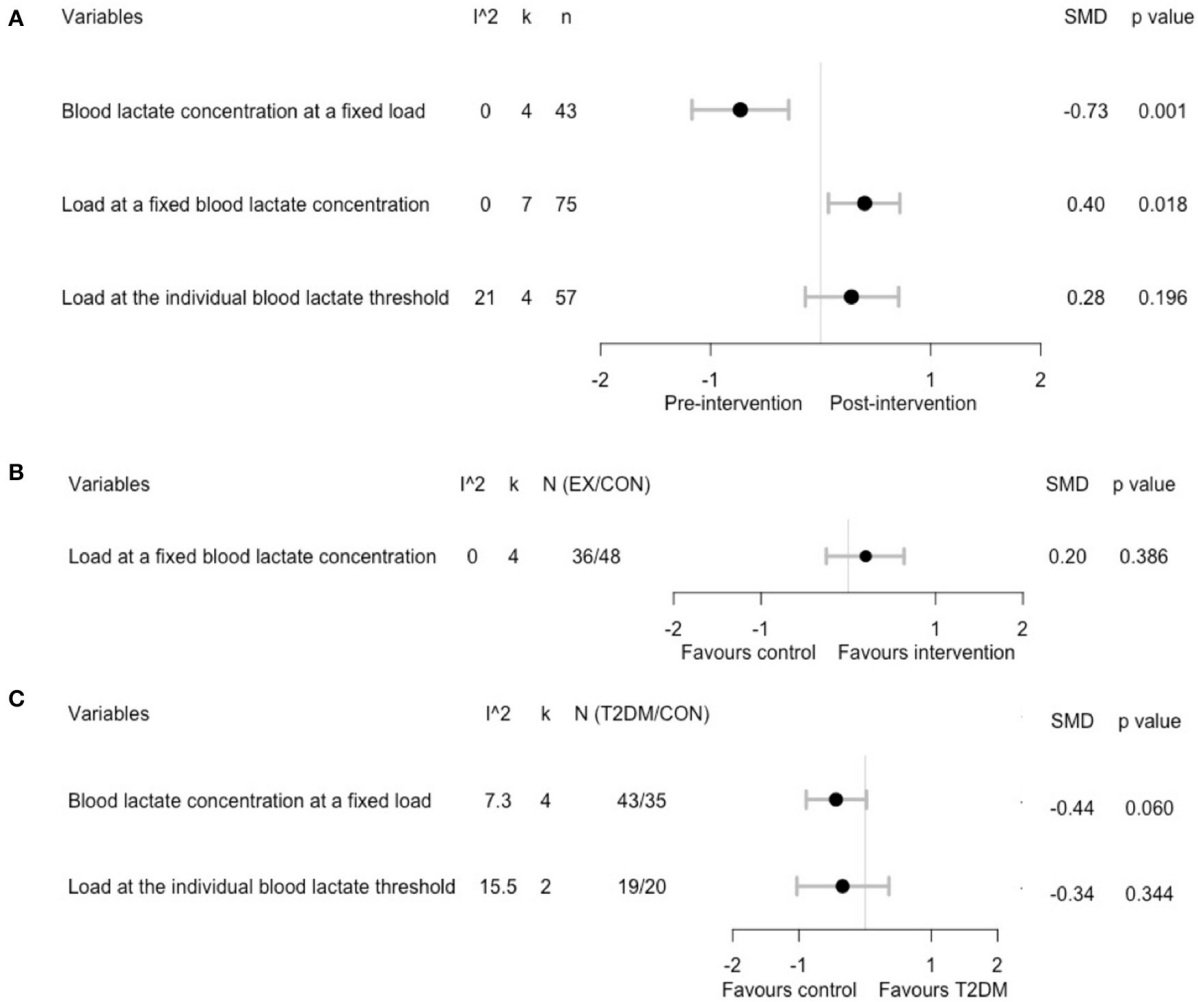
Summary estimates of the effect of chronic exercise training on blood lactate concentrations during a cardiopulmonary exercise test: **(A)** within-group analysis; **(B)** between-group analysis; **(C)** difference in relative change between T2DM patients and healthy controls.

We further assessed the effect of chronic exercise training on blood lactate metabolism during exercise and found decreased blood lactate concentrations at a fixed load (SMD = −0.73; 95% CI, −1.17 to −0.29; *p* = 0.001; *I*^2^ = 0%; Figure 3A and Supplementary Figure 3) in four studies (Schneider et al., 1987; Holton et al., 2003; Eriksen et al., 2007; de Sousa et al., 2019), while load at a fixed blood lactate concentrations was increased (SMD = 0.40; 95% CI, 0.07 to 0.72; *p* = 0.02; *I*^2^ = 0%; Figure 3A and Supplementary Figure 4) in four studies (Baum et al., 2007; Mogensen et al., 2009; Opitz et al., 2014a, 2015). No significant changes in loads at the individual blood lactate threshold were observed (SMD = 0.28; 95% CI, −0.14 to 0.71; *p* = 0.2; *I*^2^ = 21%; Figure 3A and Supplementary Figure 5) in three studies (Holton et al., 2003; Michishita et al., 2008; Støa et al., 2017). However, our sensitivity analysis by using velocity instead of %VO_2max_ showed a significant increase in loads at the individual blood lactate threshold (SMD = 0.68; 95% CI, 0.30 to 1.06; *p* < 0.001; *I*^2^ = 0%; Supplementary Figure 6), indicating the confounding effects of different measures of the blood lactate threshold. For two eligible controlled experiments (Opitz et al., 2014a, 2015), the pooled analysis showed no statistical difference in changes of load at a fixed blood lactate concentration between exercising and non-exercising T2DM groups (SMD = 0.20; 95% CI, −0.25 to 0.64; *p* = 0.39; *I*^2^ = 0%; Figure 3B and Supplementary Figure 7).

Pooling the five eligible case-control studies, the difference in changes of blood lactate concentration at a fixed load nearly reached a statistical difference between T2DM and healthy controls (SMD = −0.44; 95% CI, −0.89 to 0.02; *p* = 0.06; *I*^2^ = 0%; Figure 3C and Supplementary Figure 8). However, no significant differences were found in the change of load at the individual blood lactate threshold (SMD = −0.34; 95% CI, −1.03 to 0.36; *p* = 0.34; *I*^2^ = 16%; Figure 3C and Supplementary Figure 9) between T2DM and healthy control groups. In addition, for one case-control study (Mogensen et al., 2009) that reported load at a fixed blood lactate concentration, no significant differences were found between T2DM patients and healthy controls (SMD = 0.49; 95% CI, −0.34 to 1.32; *p* = 0.25).

Additionally, the blood lactate concentration at a fixed load assessed at baseline was higher in T2DM patients compared to healthy controls (SMD = 0.78; 95% CI, 0.31 to 1.25; *p* = 0.001; *I*^2^ = 0%; Supplementary Figure 10). However, no statistical differences were found for the baseline values of load at the individual blood lactate threshold (SMD = −0.26; 95% CI, −0.90 to 0.37; *p* = 0.41; *I*^2^ = 0%, Supplementary Figure 11). The difference in baseline values of the load at a fixed blood lactate concentration did not statistically differ between T2DM patients and healthy controls in one eligible case-control study (Mogensen et al., 2009) (SMD = −0.35; 95% CI, −1.18 to 0.48; *p* = 0.41).

### Qualitative Review

The study by Eriksen et al. (2007) was qualitatively synthesized because the raw data were unavailable. They showed that both single long-duration aerobic exercise (i.e., a single session of 30 min per day) and multiple short-duration aerobic exercises (i.e., three 10-min sessions per day) significantly decreased blood lactate concentration at a load of 90 Watts (mean differences were −0.48 and −0.43, respectively) in T2DM patients. Another study by Mogensen et al. (2009) was partially qualitative and showed that blood lactate concentrations at fixed loads (i.e., 30, 60, 90, 120, 150 Watts) were higher in T2DM patients than in healthy controls (*p* < 0.001). Blood lactate concentrations at fixed loads decreased significantly after 10 weeks of combined interval and continuous training in both groups (*p* < 0.01), but the magnitude of changes did not differ between the two groups.

## Discussion

This study assessed the effects of chronic exercise training on blood lactate metabolism at rest and during exercise in patients with T2DM. We found that chronic exercise training did not affect basal blood lactate concentrations, despite increased baseline concentrations in T2DM compared to healthy controls. However, chronic exercise training decreased blood lactate concentrations during exercise, as shown by reduced blood lactate concentration at a fixed load and/or increased load at a fixed blood lactate concentration. The current study supports the previous findings of the narrative review of Brinkmann and Brixius (2015) by means of a pooled analysis and including nine more studies.

### Effects on Basal Blood Lactate Concentrations

Five studies assessed the effects of chronic exercise training on basal blood lactate concentrations (primary outcome). However, the pooled within-group analysis for all exercising T2DM groups showed no effects on basal lactate concentrations. The reasons for this are unknown. Possibly, T2DM patients were using medications such as metformin, that are known to elevate the blood lactate level due to inhibited gluconeogenesis (Radziuk et al., 1997; Hundal et al., 2000). In fact, the use of such medication may have overridden the effect of exercise on basal blood lactate levels, but due to a lack of explicit reporting in the majority of the included studies, this remains speculative. In addition, day-to-day variations also may have contributed to the observed findings as previously a biological within-subject variation of 31% has been reported (Widjaja et al., 1999; Brinkmann and Brixius, 2015). Interestingly, we found one study (de Sousa et al., 2019) that showed a statistical decrease in basal blood lactate concentrations after 12 weeks of soccer training combined with a caloric-restricted diet intervention (i.e., reduction in energy intake of 500–1,000 kcal per day), indicating that sole exercise interventions may not be sufficient. However, these findings were not confirmed by another study (Schneider et al., 1987) that included 6 weeks of aerobic training accompanied by an American Diabetes Association diet (i.e., dietary guidelines for T2DM patients), leaving it unknown whether combined exercise and dietary interventions are superior over sole exercise training.

### Effects on Blood Lactate Metabolism During Exercise

Given that exercise leads to an increased blood lactate accumulation, the changes in blood lactate concentrations during exercise were easier to observe (Metz et al., 2005; Adeva-Andany et al., 2014). Chronic exercise training typically improves numerous aspects [e.g., oxidative capacity (Allenberg et al., 1988; Phielix et al., 2010; van Tienen et al., 2012) lactate transport and clearance (Juel et al., 2004; Opitz et al., 2014a,b; Opitz et al., 2015)] associated with normalizing lactate concentrations among T2DM patients. Indeed, we found a significant decrease in blood lactate concentration at a fixed load, while load at a fixed blood lactate concentration was increased after the training. However, the load at the individual blood lactate threshold remained unchanged. This was contrary to a meta-analysis by Londeree (1997), which reported that chronic exercise training increased load at the blood lactate threshold for sedentary subjects. This inconsistency might be explained by the different measurement methods of lactate thresholds, which was confirmed by our sensitivity analysis. Additionally, the training intensity may have been too low because current evidence in healthy populations indicates that exercise training at intensities close to or slightly above the lactate threshold was more effective to improve the load at the lactate threshold (Jones and Carter, 2000).

Furthermore, other confounders may affect the blood lactate metabolism, such as training frequency or volume (Brinkmann and Brixius, 2015), medication (i.e., metformin) (Adeva-Andany et al., 2014), obesity (Crawford et al., 2008), and hemoglobin A1c (Crawford et al., 2010). Unfortunately, in the current study we were unable to conduct further analyses for these moderators given the limited eligible studies, while the relevant data from these original articles were extracted in Table 1 and Supplementary Table 2. Future studies are encouraged to further explore which type of exercise may be most effective for improving blood lactate metabolism in T2DM patients.

### Strengths and Limitations

To the best of our knowledge, this is the first systematic review and meta-analysis that investigated the effects of chronic exercise training on blood lactate metabolism in patients with T2DM. However, this study is not without limitations.

First, the number of available studies for between-group analyses was low, thus our main conclusions were made based on the pooled SMDs of within-group comparisons, which are vulnerable to many threats of internal validity (Littell et al., 2008). Second, this study did not focus on the mechanisms that are responsible for changes in blood lactate metabolism. Future research may investigate the potential differences in physiological mechanisms in blood lactate metabolism between patients with T2DM and healthy individuals. Third, the definition of chronic exercise training might be variable from the duration and type of exercise intervention. Last, our findings should be interpreted with caution due to the limited numbers of included studies and participants, as well as the low quality of evidence (e.g., high heterogeneity of study designs).

In conclusion, although measures of blood lactate concentrations are a standard assessment in exercise physiology related studies, we revealed a dramatic lack of studies assessing the blood lactate metabolism in T2DM. Nevertheless, our findings indicate that chronic exercise training can improve blood lactate metabolism during exercise among T2DM patients, by decreasing blood lactate concentration at a fixed load or increasing load at a fixed blood lactate concentration. However, the observed improvements in the lactate metabolism during exercise did not translate into reductions in basal blood lactate concentrations. Future studies are needed to investigate the mechanisms underlying this lack of effect of chronic exercise training on the basal lactate metabolism. Elucidating these effects is of clinical importance for T2DM patients to improve glycemic control and to avoid side effects of the long-term elevated basal lactate level on the kidney.

## Data Availability Statement

The raw data supporting the conclusions of this article will be made available by the authors, without undue reservation.

## Author Contributions

TZ initiated and designed the study, selected studies, extracted data, assessed the quality of included studies, performed data analysis, and drafted the manuscript. SL selected studies, extracted data, assessed the quality of included studies, contributed to the interpretation of the results, and edited the manuscript. SC initiated and designed the study, contributed to the interpretation of the results, and edited the manuscript. NF contacted authors for original data, contributed to the interpretation of the results, and edited the manuscript. MS and XW contributed to the interpretation of the results and edited the manuscript. All authors have read and approved the final version of the manuscript and agreed with the order of presentation of the authors.

## Conflict of Interest

The authors declare that the research was conducted in the absence of any commercial or financial relationships that could be construed as a potential conflict of interest.

## Supplementary Material

Supplementary Figure 1 Sensitivity analysis for within-group analysis of the effect of chronic exercise training on basal blood lactate concentrations.

Supplementary Figure 2 Difference in baseline values of basal blood lactate concentrations between T2DM patients and healthy controls.

Supplementary Figure 3 Within-group analysis regarding the effect of chronic exercise on blood lactate concentration at a fixed load.

Supplementary Figure 4 Within-group analysis regarding the effect of chronic exercise on load at a fixed blood lactate concentration.

Supplementary Figure 5 Within-group analysis regarding the effect of chronic exercise on load at the individual blood lactate threshold.

Supplementary Figure 6 Sensitivity analysis of the effect of chronic exercise training on load at the individual blood lactate threshold by using different measurements of lactate threshold.

Supplementary Figure 7 Between-group analysis regarding the effect of chronic exercise on load at a fixed blood lactate concentration.

Supplementary Figure 8 Difference in relative change of blood lactate concentration at a fixed load between T2DM patients and healthy controls.

Supplementary Figure 9 Difference in relative change of load at the individual blood lactate threshold between T2DM patients and healthy controls.

Supplementary Figure 10 Difference in baseline values of blood lactate concentration at a fixed load between T2DM patients and healthy controls.

Supplementary Figure 11 Difference in baseline values of load at the individual blood lactate threshold between T2DM patients and healthy controls.

Supplementary Table 1 Search strategy.

Supplementary Table 2 Medication status of participants.

Supplementary Table 3 GRADE evidence profile for within-group analyses.

Supplementary Table 4 Quality assessment of included studies.
