# Supplementary figures and images for: Effect of Chronic Exercise Training on Blood Lactate Metabolism Among Patients With Type 2 Diabetes Mellitus: A Systematic Review and Meta-Analysis
Source: Front Physiol. 2021 Mar 11;12:652023. doi: 10.3389/fphys.2021.652023 (PMC7992008; doi:10.3389/fphys.2021.652023)

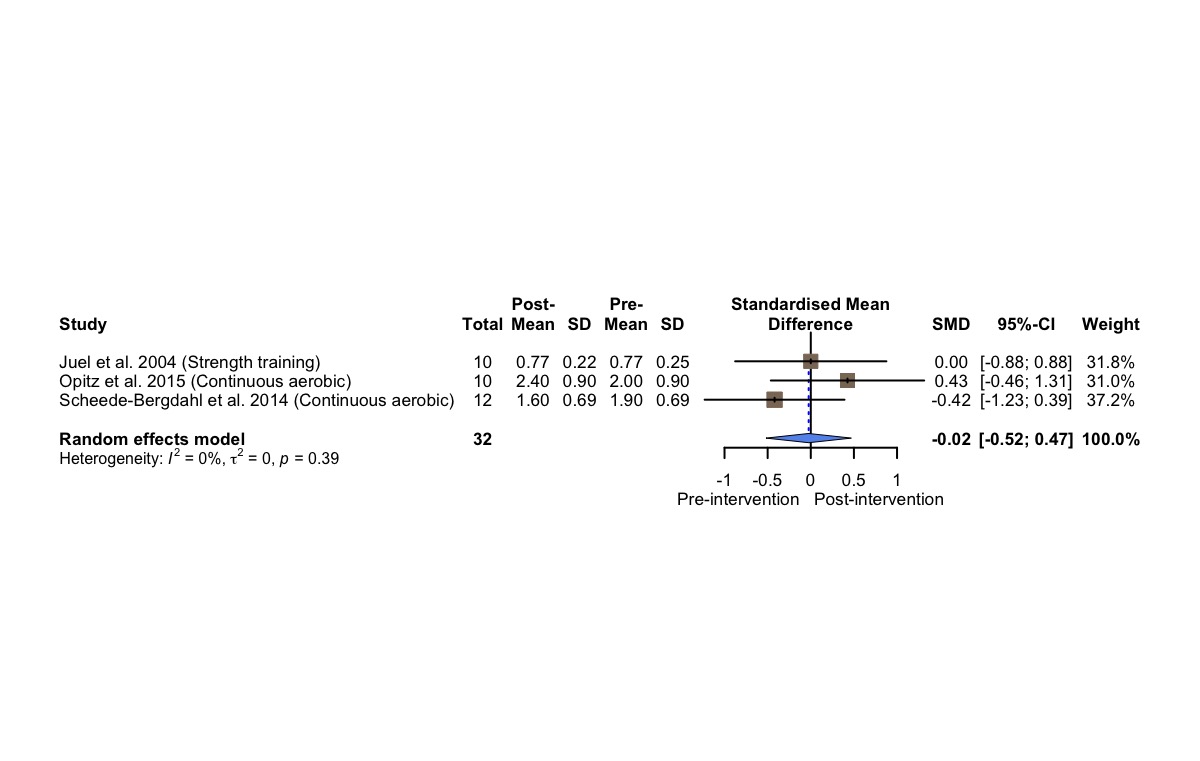

Supplement: Supplementary Figure 1 — Sensitivity analysis for within-group analysis of the effect of chronic exercise training on basal blood lactate concentrations. [file Image_1.JPEG]

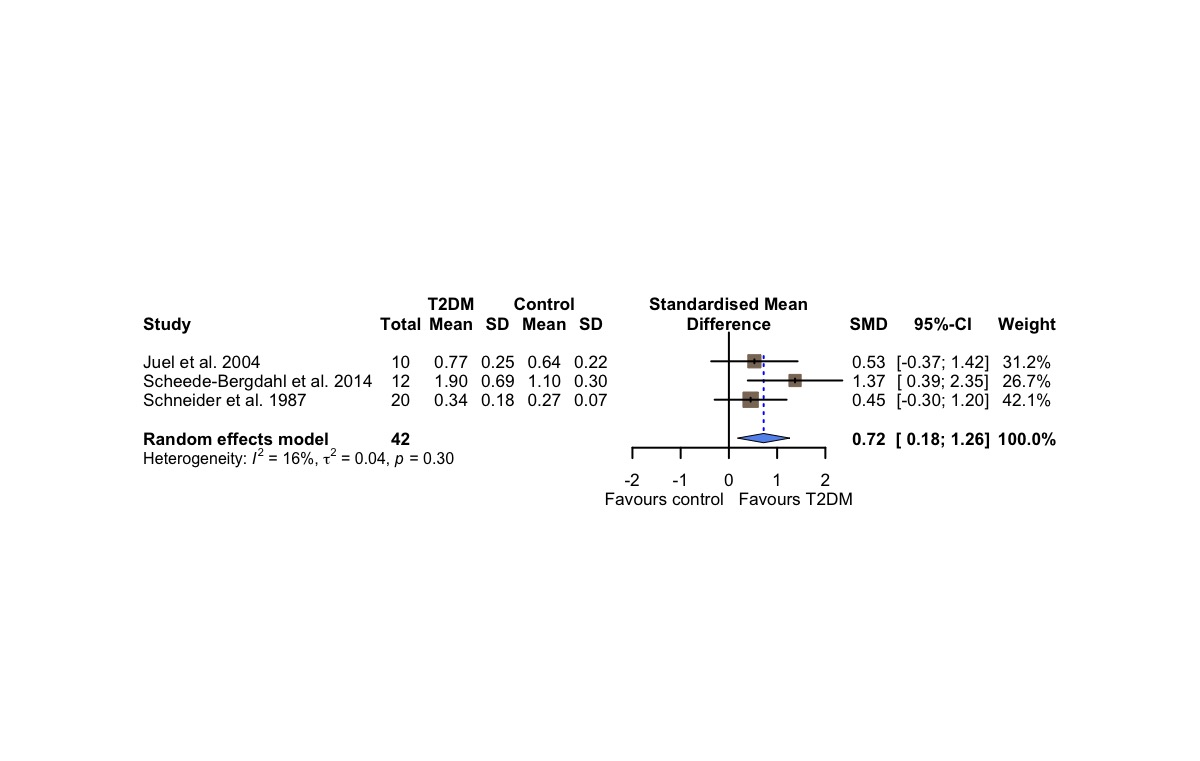

Supplement: Supplementary Figure 2 — Difference in baseline values of basal blood lactate concentrations between T2DM patients and healthy controls. [file Image_2.JPEG]

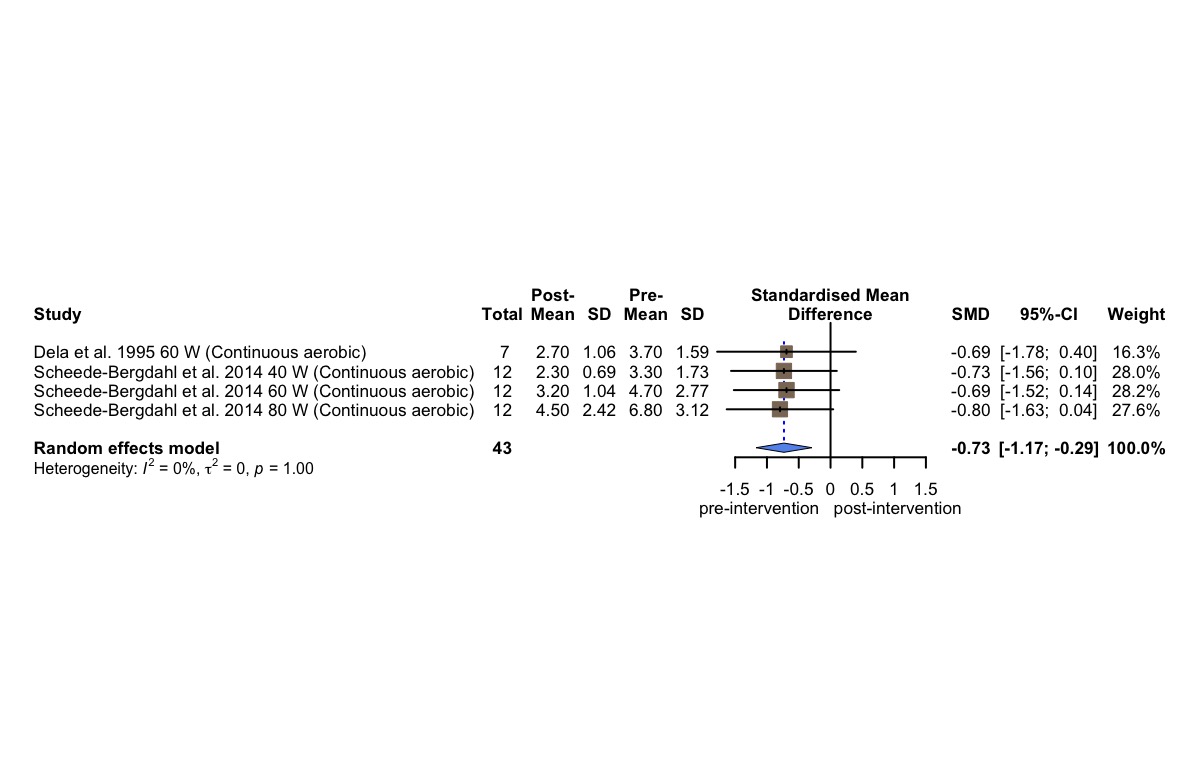

Supplement: Supplementary Figure 3 — Within-group analysis regarding the effect of chronic exercise on blood lactate concentration at a fixed load. [file Image_3.JPEG]

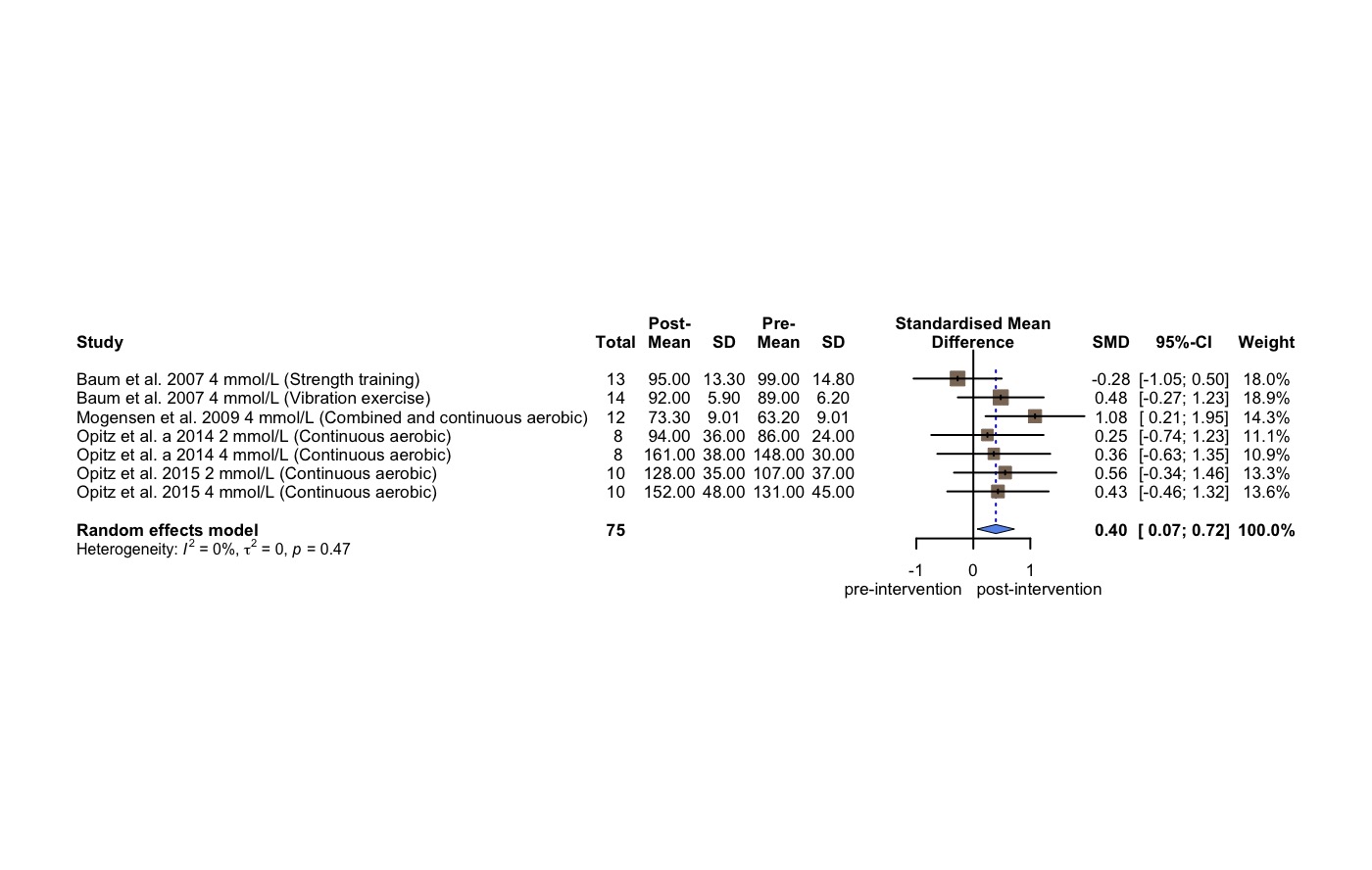

Supplement: Supplementary Figure 4 — Within-group analysis regarding the effect of chronic exercise on load at a fixed blood lactate concentration. [file Image_4.JPEG]

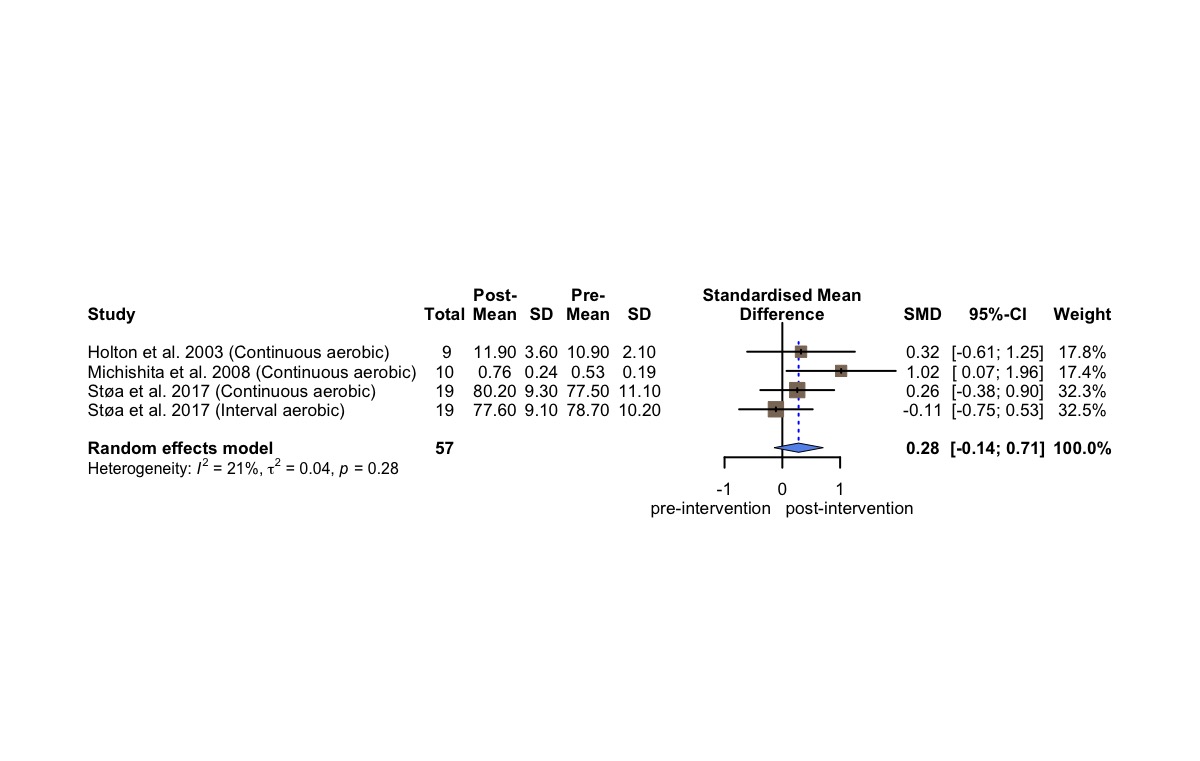

Supplement: Supplementary Figure 5 — Within-group analysis regarding the effect of chronic exercise on load at the individual blood lactate threshold. [file Image_5.JPEG]

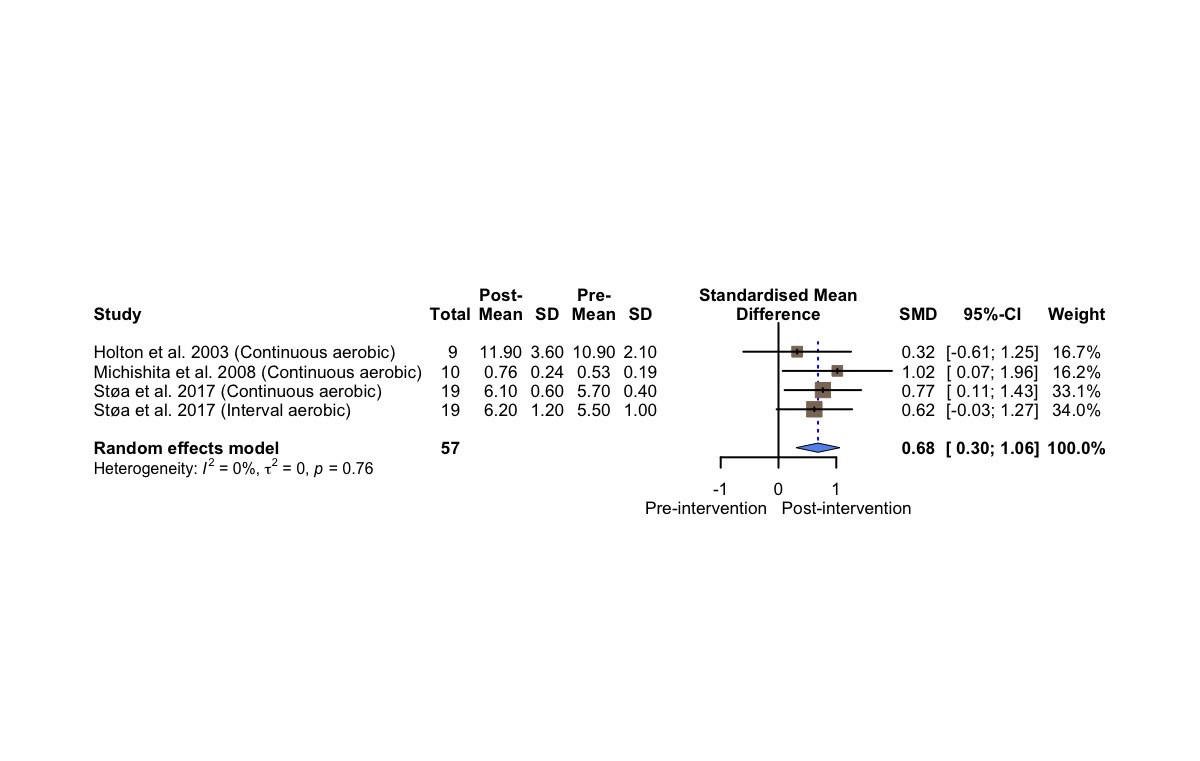

Supplement: Supplementary Figure 6 — Sensitivity analysis of the effect of chronic exercise training on load at the individual blood lactate threshold by using different measurements of lactate threshold. [file Image_6.JPEG]

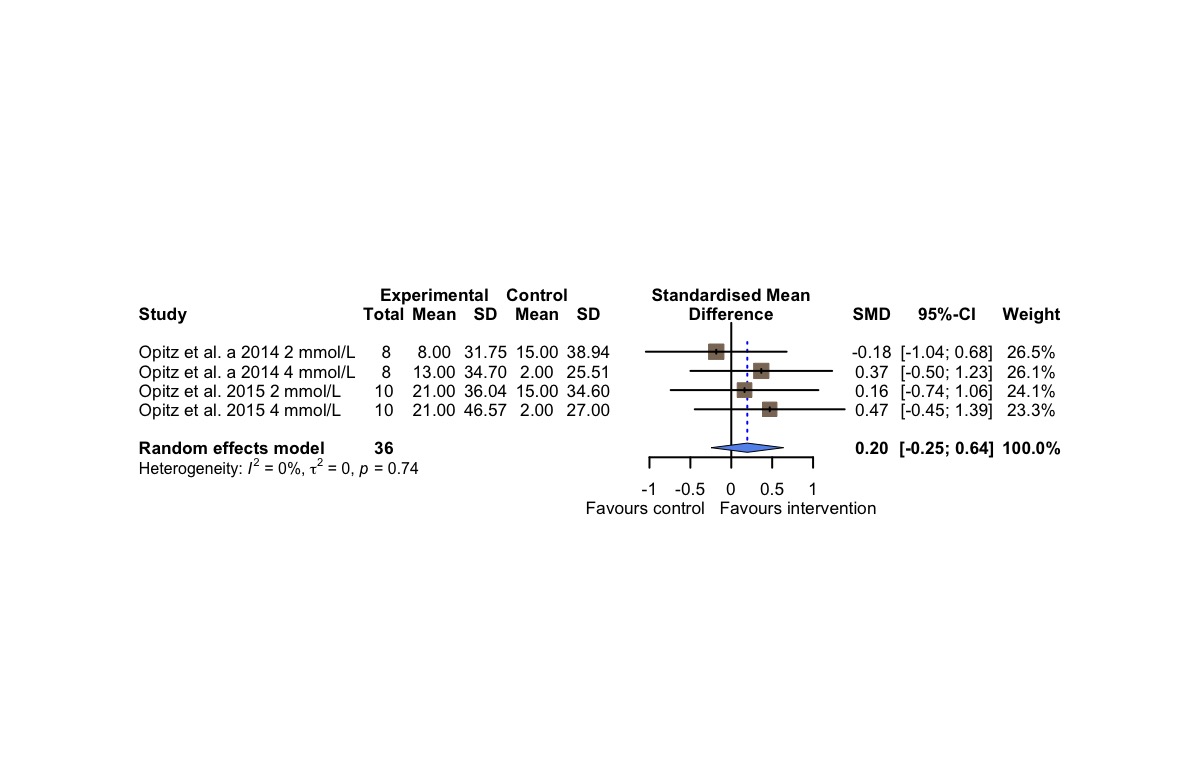

Supplement: Supplementary Figure 7 — Between-group analysis regarding the effect of chronic exercise on load at a fixed blood lactate concentration. [file Image_7.JPEG]

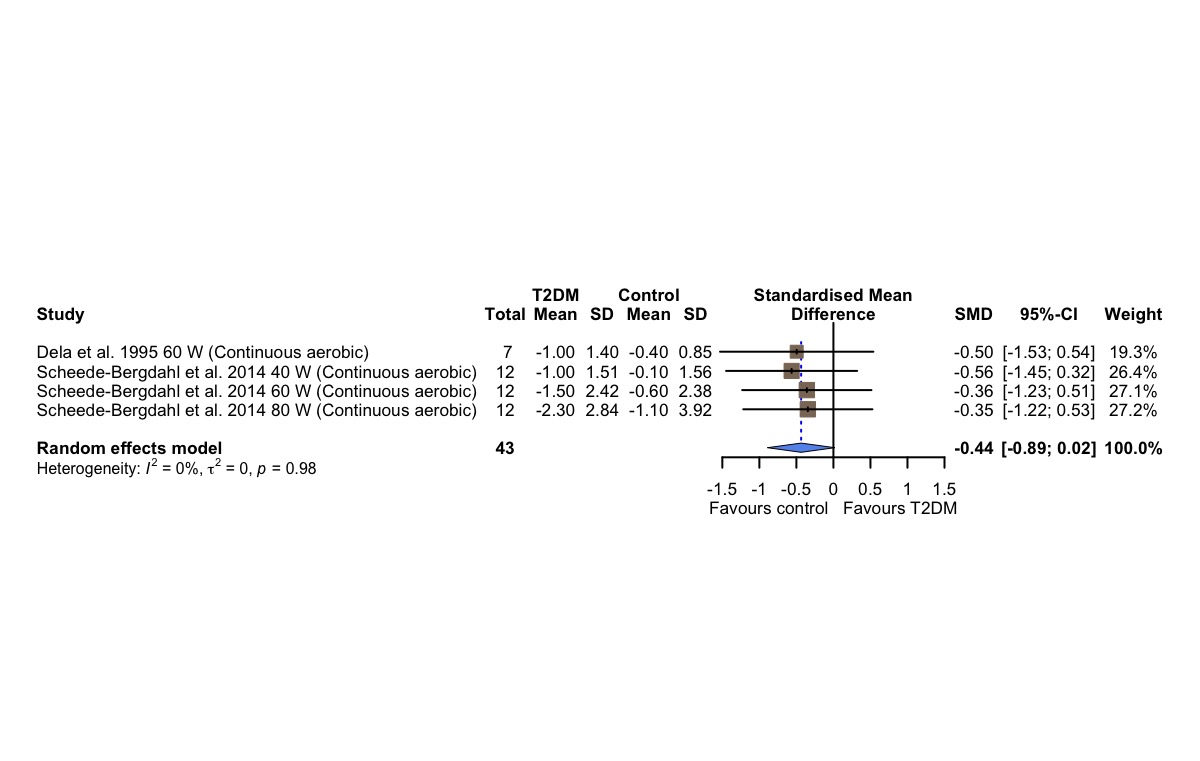

Supplement: Supplementary Figure 8 — Difference in relative change of blood lactate concentration at a fixed load between T2DM patients and healthy controls. [file Image_8.JPEG]

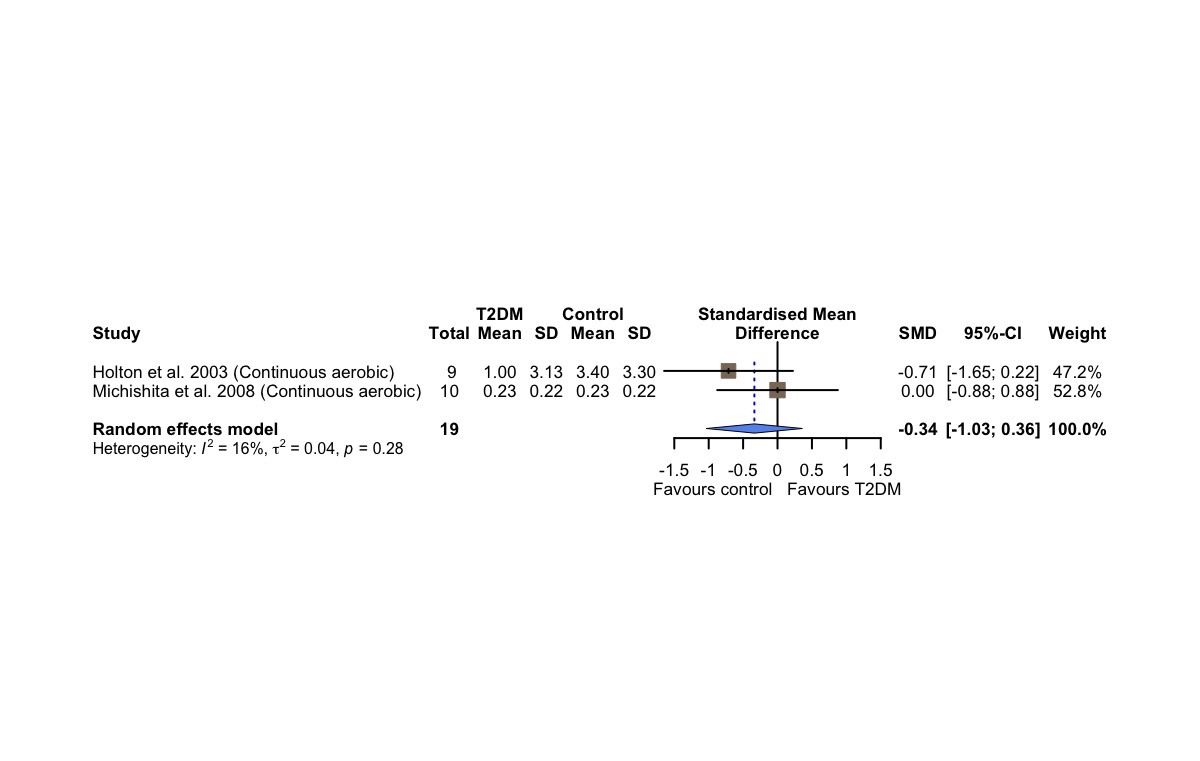

Supplement: Supplementary Figure 9 — Difference in relative change of load at the individual blood lactate threshold between T2DM patients and healthy controls. [file Image_9.JPEG]

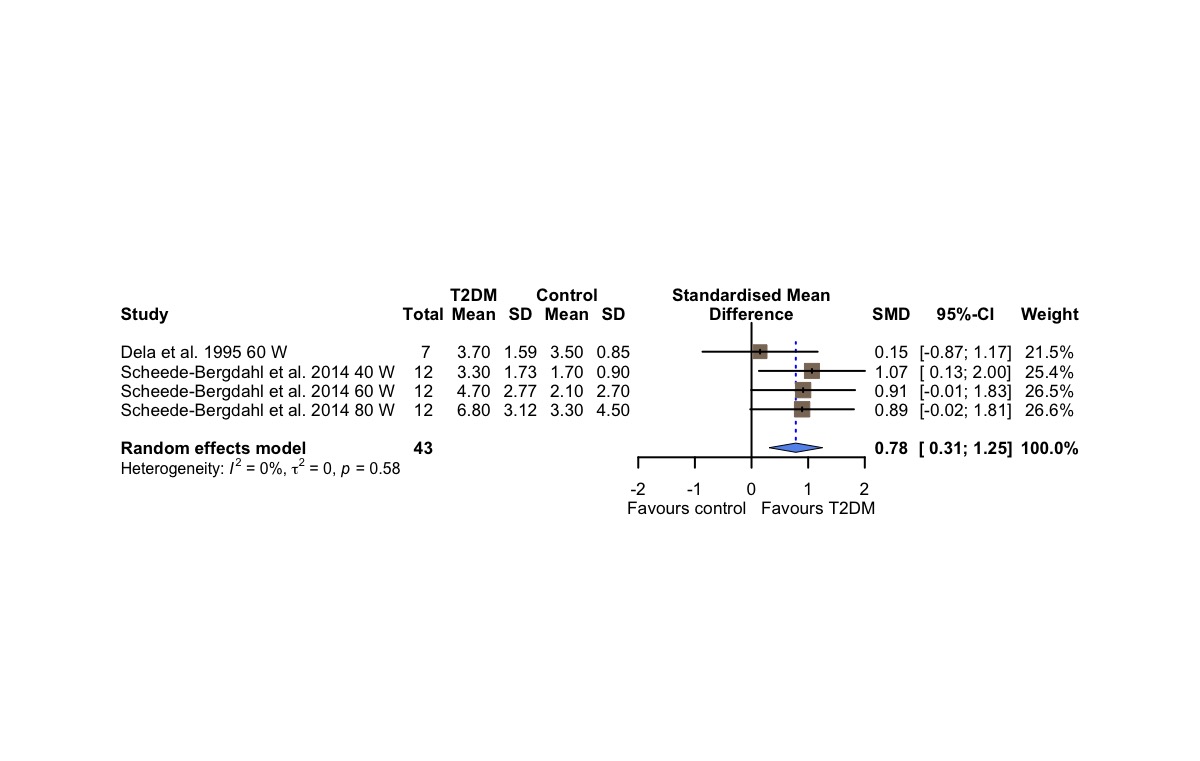

Supplement: Supplementary Figure 10 — Difference in baseline values of blood lactate concentration at a fixed load between T2DM patients and healthy controls. [file Image_10.JPEG]

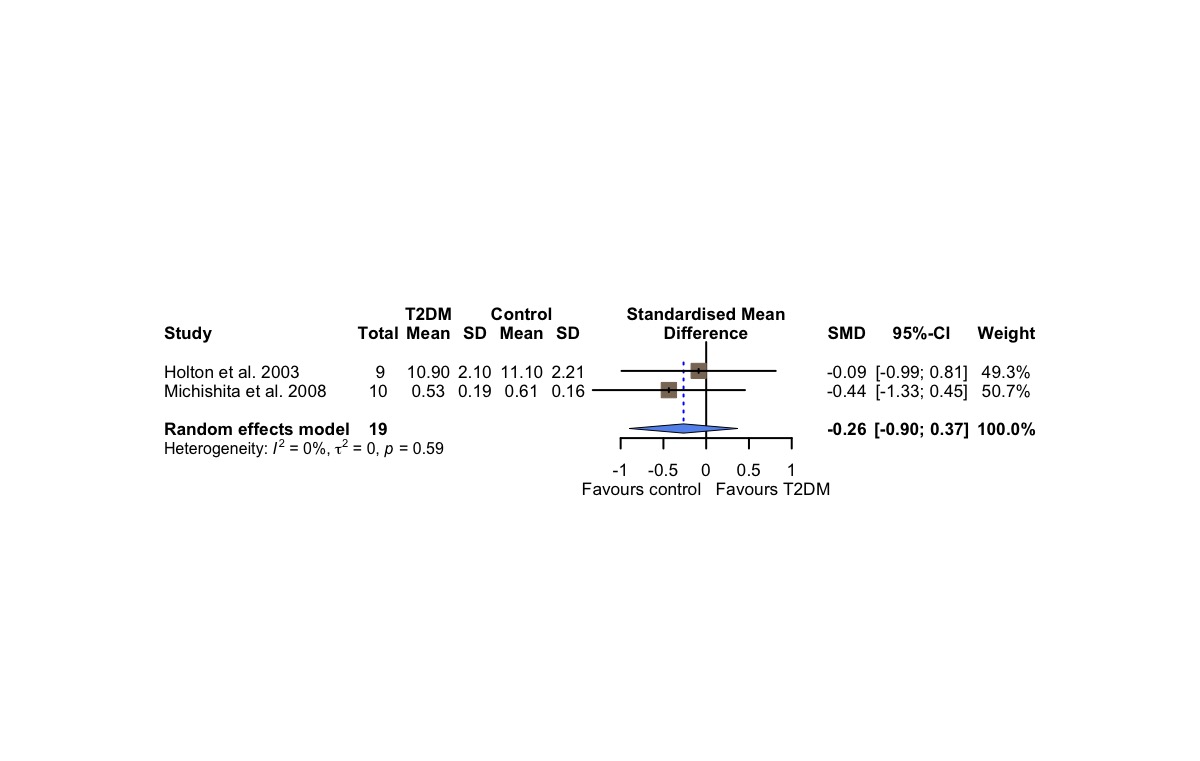

Supplement: Supplementary Figure 11 — Difference in baseline values of load at the individual blood lactate threshold between T2DM patients and healthy controls. [file Image_11.JPEG]
