# Supplementary material for: Effect of Chronic Exercise Training on Blood Lactate Metabolism Among Patients With Type 2 Diabetes Mellitus: A Systematic Review and Meta-Analysis
Source: Front Physiol. 2021 Mar 11;12:652023. doi: 10.3389/fphys.2021.652023 (PMC7992008; doi:10.3389/fphys.2021.652023)
Supplement: Supplementary Table 1 — Search strategy. [file Table_1.DOCX]

**Table S1.** Search strategy

| Databases | Search strategy |
| --- | --- |
| PubMed (MedLine) | (("Humans"[Mesh]) AND ((("Exercise"[Mesh]) OR ("Sports"[Mesh]) OR ("Exercise Therapy"[Mesh])) AND (("Diabetes Mellitus, Type 2"[Mesh]) OR ("Insulin Resistance"[Mesh]))) |
| Embase | ('human'/exp OR 'human') AND ('exercise'/exp OR 'exercise' OR 'kinesiotherapy'/exp OR 'kinesiotherapy') AND ('non insulin dependent diabetes mellitus'/exp OR 'non insulin dependent diabetes mellitus' OR 'insulin resistance'/exp OR 'insulin resistance') AND [article]/lim AND [english]/lim |
| Web of Science | TS=(humans) AND TS=(exercise OR "exercise therapy" OR sports) AND TS=("diabetes mellitus type 2" OR diabetes OR "insulin resistance" OR "glycemic control")  Indexes=SCI-EXPANDED, SSCI, A&HCI, ESCI Timespan=All years |
