# Supplementary material for: Effect of Chronic Exercise Training on Blood Lactate Metabolism Among Patients With Type 2 Diabetes Mellitus: A Systematic Review and Meta-Analysis
Source: Front Physiol. 2021 Mar 11;12:652023. doi: 10.3389/fphys.2021.652023 (PMC7992008; doi:10.3389/fphys.2021.652023)
Supplement: Supplementary Table 2 — Medication status of participants. [file Table_2.DOCX]

**Table S2.** Medication status of participants

| No. | Reference | Medication for diabetes participants, n |
| --- | --- | --- |
| 1 | Schneider et al. 1987 | No medication  (according manuscript) |
| 2 | Dela et al. 1995 | Glipiazide 3x80mg, 1 Metformin 3x1g, 1 Chlorpropamide 250mg, 1 Glipizide 2x3.5mg, 1 Metformin 2x500mg, 1 Tolbutamide 2x500mg, 1 Diltiazem 2x120mg, 1 |
| 3 | Holton et al. 2003 | Not shown. |
| 4 | Juel et al. 2004 | Tolbutamide 1000mg/d, 2 Glibenclamide 7mg/d, 1 Metformin 1700mg/d, 1 Amlodipin 5mg/d, 1 Cerivastatin 200ug/d, 1 |
| 5 | Baum et al 2007 | Not shown. |
| 6 | Eriksen et al.2007 | Not shown. |
| 7 | Michishita et al. 2008 | No medication (according manuscript) |
| 8 | Mogensen et al. 2009 | Metformin, 6 Sulphonyluria, 1 Metformin + sulphonyluria, 4 Antihypertensives, 7 Lipid-lowering agents, 6 |
| 9 | Opitz et al. 2014 | Not shown. |
| 10 | Scheede-Bergdahl et al. 2014 | Metformin, 2 Metformin + sulfonylurea, 5 Statins, 2 Ace inhibitor, 2 Beta-blocker, 1 Anti-diuretics, 2 |
| 11 | Opitz et al. 2015 | Not shown. |
| 12 | Støa et al. 2017 | Biguanides (metformin or glucophage), 9/12 † Sulfonylurea medications, 3/6 † DPP-4 inhibitors, 2/2 † GLP-1 analog, 0/2 † Pioglitazone, 0/1 † Insulin, 2/3 † Hypertension, 10/10 † Cholesterol, 12/9 † |
| 13 | de Sousa et al. 2019 | Antihypertensive agents, 24  Statins, 16 Metformin, 17  Metformin + sulphonylurea, 23 |
