# Supplementary material for: Effect of Chronic Exercise Training on Blood Lactate Metabolism Among Patients With Type 2 Diabetes Mellitus: A Systematic Review and Meta-Analysis
Source: Front Physiol. 2021 Mar 11;12:652023. doi: 10.3389/fphys.2021.652023 (PMC7992008; doi:10.3389/fphys.2021.652023)
Supplement: Supplementary Table 3 — GRADE evidence profile for within-group analyses. [file Table_3.DOCX]

**Table S3.** GRADE evidence profile for within-group analyses.

| **Certainty assessment** | | | | | | | **№ of patients** | **Absolute Effect (95% CI)** | **Certainty** |
| --- | --- | --- | --- | --- | --- | --- | --- | --- | --- |
| **№ of studies** | **Study design** | **Risk of bias** | **Inconsistency** | **Indirectness** | **Imprecision** | **Other considerations** |  |  |  |
| **Basal blood lactate concentration** | | | | | | | | | |
| 5 | observational studies | not serious | not serious | not serious | not serious | none | 71 | SMD **0.2 SD lower** (0.55 lower to 0.16 higher) | ⨁⨁◯◯ LOW |
| **Blood lactate concentration at a fixed load** | | | | | | | | | |
| 4 | observational studies | serious ^a^ | not serious | not serious | not serious | none | 43 | SMD **0.73 SD higher** (1.17 lower to 0.29 higher) | ⨁◯◯◯ VERY LOW |
| **Load at a fixed blood lactate concentration** | | | | | | | | | |
| 7 | observational studies | not serious | not serious | not serious | not serious | none | 75 | SMD **0.4 SD higher** (0.07 higher to 0.72 higher) | ⨁⨁◯◯ LOW |
| **Load at the individual blood lactate threshold** | | | | | | | | | |
| 4 | observational studies | not serious | not serious | not serious | not serious | none | 57 | SMD **0.28 SD higher** (0.14 lower to 0.71 higher) | ⨁⨁◯◯ LOW |

**CI:** Confidence interval; **SMD:** Standardized mean difference

#### Explanations

a. This finding was not supported by at studies with excellent or good quality.
