# Supplementary material for: Effect of Chronic Exercise Training on Blood Lactate Metabolism Among Patients With Type 2 Diabetes Mellitus: A Systematic Review and Meta-Analysis
Source: Front Physiol. 2021 Mar 11;12:652023. doi: 10.3389/fphys.2021.652023 (PMC7992008; doi:10.3389/fphys.2021.652023)
Supplement: Supplementary Table 4 — Quality assessment of included studies. [file Table_4.docx]

**Table S4.** Quality assessment of included studies

| No. | Study | Reporting | | | | | | | | | | External Validity | | | Internal validity – bias | | | | | | | Internal validity‐ confounding | | | | | | Power | Total score |
| --- | --- | --- | --- | --- | --- | --- | --- | --- | --- | --- | --- | --- | --- | --- | --- | --- | --- | --- | --- | --- | --- | --- | --- | --- | --- | --- | --- | --- | --- |
|  |  | 1 | 2 | 3 | 4 | 5 | 6 | 7 | 8 | 9 | 10 | 11 | 12 | 13 | 14 | 15 | 16 | 17 | 18 | 19 | 20 | 21 | 22 | 23 | 24 | 25 | 26 | 27 |  |
| 1 | Schneider et al. 1987 | 0 | 1 | 1 | 1 | 2 | 1 | 1 | 0 | 1 | 0 | 0 | 0 | 1 | 0 | 0 | 1 | 1 | 1 | 1 | 1 | 0 | 0 | 0 | 0 | 0 | 1 | 0 | 15 |
| 2 | Dela et al. 1995 | 1 | 1 | 1 | 1 | 2 | 1 | 1 | 0 | 1 | 0 | 0 | 0 | 0 | 0 | 0 | 1 | 1 | 1 | 1 | 1 | 0 | 0 | 0 | 0 | 0 | 1 | 0 | 15 |
| 3 | Holton et al. 2003 | 1 | 1 | 1 | 1 | 2 | 1 | 1 | 0 | 1 | 0 | 1 | 1 | 1 | 0 | 0 | 1 | 1 | 1 | 1 | 1 | 0 | 0 | 0 | 0 | 0 | 1 | 0 | 18 |
| 4 | Juel et al. 2004 | 1 | 1 | 0 | 1 | 1 | 1 | 1 | 0 | 1 | 1 | 0 | 1 | 1 | 0 | 0 | 1 | 1 | 1 | 1 | 1 | 0 | 0 | 0 | 0 | 0 | 1 | 0 | 16 |
| 5 | Baum et al. 2007 | 1 | 1 | 1 | 1 | 1 | 1 | 1 | 1 | 1 | 0 | 0 | 0 | 1 | 0 | 0 | 1 | 1 | 1 | 1 | 1 | 0 | 0 | 1 | 0 | 0 | 1 | 0 | 17 |
| 6 | Eriksen et al. 2007 | 1 | 1 | 1 | 1 | 2 | 1 | 1 | 0 | 1 | 1 | 1 | 1 | 0 | 0 | 0 | 1 | 0 | 1 | 1 | 1 | 1 | 0 | 1 | 0 | 0 | 0 | 0 | 18 |
| 7 | Michishita et al. 2008 | 1 | 1 | 1 | 1 | 2 | 1 | 1 | 0 | 1 | 0 | 1 | 1 | 0 | 0 | 0 | 1 | 1 | 1 | 1 | 1 | 1 | 0 | 0 | 0 | 1 | 1 | 0 | 19 |
| 8 | Mogensen et al. 2009 | 1 | 1 | 1 | 1 | 2 | 1 | 1 | 0 | 1 | 1 | 1 | 1 | 0 | 0 | 0 | 1 | 1 | 1 | 1 | 1 | 0 | 0 | 0 | 0 | 1 | 1 | 1 | 20 |
| 9 | Opitz et al. 2014a | 1 | 1 | 1 | 1 | 2 | 1 | 1 | 0 | 1 | 0 | 1 | 1 | 1 | 0 | 0 | 1 | 1 | 1 | 1 | 1 | 0 | 0 | 0 | 0 | 1 | 1 | 0 | 19 |
| 10 | Scheede-Bergdahl et al. 2014 | 1 | 1 | 0 | 1 | 2 | 1 | 1 | 0 | 1 | 0 | 0 | 0 | 0 | 0 | 0 | 1 | 1 | 1 | 0 | 1 | 0 | 0 | 0 | 0 | 0 | 1 | 0 | 13 |
| 11 | Opitz et al. 2015 | 1 | 1 | 1 | 1 | 2 | 1 | 1 | 0 | 1 | 1 | 1 | 1 | 1 | 0 | 0 | 1 | 1 | 1 | 1 | 1 | 0 | 0 | 0 | 0 | 1 | 1 | 0 | 20 |
| 12 | Støa et al. 2017 | 1 | 1 | 1 | 1 | 2 | 1 | 1 | 0 | 1 | 1 | 1 | 1 | 1 | 0 | 0 | 1 | 1 | 1 | 1 | 1 | 1 | 0 | 0 | 0 | 1 | 1 | 0 | 21 |
| 13 | de Sousa et al. 2019 | 1 | 1 | 1 | 1 | 2 | 1 | 1 | 0 | 1 | 0 | 0 | 1 | 1 | 0 | 0 | 1 | 1 | 1 | 1 | 1 | 0 | 0 | 1 | 1 | 1 | 0 | 0 | 19 |
